# Biofilm formation by multidrug resistant *Enterobacteriaceae* strains isolated from solid organ transplant recipients

**DOI:** 10.1038/s41598-019-45060-y

**Published:** 2019-06-20

**Authors:** José Ramos-Vivas, Itziar Chapartegui-González, Marta Fernández-Martínez, Claudia González-Rico, Jesús Fortún, Rosa Escudero, Francesc Marco, Laura Linares, Miguel Montejo, Maitane Aranzamendi, Patricia Muñoz, Maricela Valerio, Jose María Aguado, Elena Resino, Irene Gracia Ahufinger, Aurora Paz Vega, Luis Martínez-Martínez, María Carmen Fariñas, The ENTHERE Study Group, the Group for Study of Infection in Transplantation of the Spanish Society of Infectious Diseases and Clinical Microbiology (GESITRA-SEIMC) and the Spanish Network for Research in Infectious Diseases (REIPI), Juan Carlos Ruiz San Millán, Emilio Rodrigo, Fernando Casafont Morencos, Emilio Fabrega, Antonio Cuadrado, Concepción Fariñas-Alvarez, Mónica Gozalo, Francisco Arnaíz de las Revillas, Pilar Martín Dávila, Adolfo Martínez, Patricia Ruíz Garbajosa, Asunción Moreno, Marta Bodro, María Fernanda Solano, María José Blanco, Javier Nieto, Marina Machado, María Olmedo, Sara Rodríguez Fernández, Cristina Rincón Sanz, Teresa Vicente Range, Caroline Agnelli Bento, Alicia Galar Recalde, Alia Eworo, Fernando Anaya Fernández-Lomana, María Luisa Rodríguez-Ferrero, Luis Alberto Sánchez Cámara, Fernando Chaves, Julián de la Torre Cisneros

**Affiliations:** 1 Instituto de Investigación Valdecilla-IDIVAL, Avd. Cardenal Herrera Oria, 39011 Santander, Spain; 2 Service of Microbiology, Hospital Universitario Marqués de Valdecilla, Avd. Valdecilla, 39008 Santander, Spain; 3 Infectious Diseases Unit. Hospital Universitario Marqués de Valdecilla, Santander, Spain. Avd. Valdecilla, 39008 Santander, Spain; 4 Infectious Diseases Department, Hospital Universitario Ramón y Cajal, Ctra. Colmenar Viejo, km. 9, 100, 28034 Madrid, Spain; 5 Service of Microbiology, Hospital Clínic-IDIBAPS, Universidad de Barcelona, Carrer de Villarroel, 170, 08036 Barcelona, Spain; 6 Infectious Diseases Service, Hospital Clínic-IDIBAPS, Universidad de Barcelona, Carrer de Villarroel, 170, 08036 Barcelona, Spain; 7 Infectious Diseases Unit, Hospital Universitario Cruces, Plaza de Cruces, S/N, 48903 Baracaldo, Vizcaya Spain; 8 Service of Microbiology, Hospital Universitario Cruces, Plaza de Cruces, S/N, 48903 Baracaldo, Vizcaya Spain; 9 Clinical Microbiology and Infectious Diseases, Hospital General Universitario Gregorio Marañón, Calle del Dr. Esquerdo, 46, 28007 Madrid, Spain; 10 Infectious Diseases Unit, Hospital Universitario 12 de Octubre, Av. Córdoba, s/n, 28004 Madrid, Spain; 11 Service of Microbiology, Hospital Universitario Reina Sofía, Av. Menéndez Pidal, s/n, 14004 Córdoba, Spain; 12 Infectious Diseases Unit, Hospital Universitario Reina Sofía, Av. Menéndez Pidal, s/n, 14004 Córdoba, Spain; 13 Nephrology Department, Hospital Universitario Marqués de Valdecilla, Santander, Spain; 14 Liver Unit, Hospital Universitario Marqués de Valdecilla, Santander, Spain; 15 Quality Unit, Hospital Universitario Marqués de Valdecilla, Santander, Spain; 16 Transplant Coordination, Hospital Universitario Ramón y Cajal, Madrid, Spain; 17 Service of Microbiology, Hospital Universitario Ramón y Cajal, Madrid, Spain; 18 Department of Nephrology, Hospital General Universitario Gregorio Marañón, Madrid, Spain; 19 Service of Microbiology, Hospital Universitario 12 de Octubre, Madrid, Spain

**Keywords:** Medical research, Clinical microbiology

## Abstract

Solid organ transplant (SOT) recipients are especially at risk of developing infections by multidrug resistant bacteria (MDR). In this study, the biofilm-forming capability of 209 MDR strains (*Escherichia coli* n = 106, *Klebsiella pneumoniae* n = 78, and *Enterobacter* spp. n = 25) isolated from rectal swabs in the first 48 hours before or after kidney (93 patients), liver (60 patients) or kidney/pancreas transplants (5 patients) were evaluated by using a microplate assay. Thirty-nine strains were isolated before transplant and 170 strains were isolated post-transplant. Overall, 16% of *E. coli* strains, 73% of *K. pneumoniae* strains and 4% *Enterobacter* strains showed moderate or strong biofilm production. Nine strains isolated from infection sites after transplantation were responsible of infections in the first month. Of these, 4 *K. pneumoniae*, 1 *E. coli* and 1 *Enterobacter* spp. strains isolated pre-transplant or post-transplant as colonizers caused infections in the post-transplant period. Our results suggest that *in vitro* biofilm formation could be an important factor for adhesion to intestine and colonization in MDR *K. pneumoniae* strains in SOT recipients, but this factor appears to be less important for MDR *E. coli* and *Enterobacter* spp.

## Introduction

Solid organ transplant (SOT) recipients have an increased risk of developing bacterial infections because they receive long immunosuppressive therapy to avoid rejection. Furthermore, SOT recipients are especially at risk of developing infections by bacteria with intrinsic or acquired antimicrobial resistance as they are frequently exposed to antibiotics in healthcare settings^1–3^. Currently, the increasing prevalence of multi-drug resistant (MDR) Gram-negative pathogens, such as extended-spectrum β-lactamase-producing (ESBLs) *Enterobacteriaceae* strains, strains with overproduction of intrinsic chromosomal AmpC β-lactamases^4,5^, and carbapenem-resistant *K. pneumoniae* and *E. coli* strains, are of particular concern in SOT recipients.

Important risk factors for nosocomial infections in SOT recipients are, among others, the use of catheters, hospitalization in intensive care units, and pre-transplant faecal carriage of multidrug-resistant isolates^6,7^.

The *Enterobacteriaceae* family is a heterogeneous group of Gram-negative bacteria, whose natural habitat is the intestinal tract of humans and animals. Among the hospital-acquired infections due to *Enterobacteriaceae*, urinary tract infections (UTIs) are the most common and lower respiratory tract and bloodstream infections are the most lethal^8–10^. Virulence factors in *Enterobacteriaceae* include different adhesins, hemolysin production, serum resistance, and biofilm formation. These factors, especially the ability to form biofilms in the human intestine may contribute to gut colonization and have a great impact on the function of the intestinal microbiome and its interactions with the host^11–19^.

A biofilm is often defined as a structured community of microorganisms enclosed in a self-produced matrix and adherent to an inert or living surface. Growth in biofilms enhances the survival of bacterial populations in hospital settings and inside patients, increasing the probability of causing nosocomial infections^20–22^. Also, several investigations have revealed that horizontal gene transfer is connected with biofilm formation^23,24^.

Previous studies showed a relationship between the presence of antimicrobial resistance genes and biofilm formation in *E. coli* and *Klebsiella* strains isolated from patients with different infections^14,25,26^, however, the correlation between antibiotic resistance and biofilm formation for most pathogens (i.e. *Enterobacter* spp.) remains unclear. The goal of present study, which is part of a wider research project (The ENTHERE study), was to analyse the capacity to form biofilm of 209 *Enterobacteriaceae* strains isolated from pre- or post-transplant patients. This investigation could contribute to a better understanding the relationship between adherence capability, antimicrobial resistance, and pathogenicity of MDR bacteria in SOT recipients.

## Results

### Antibiotic resistance genes

The most common genes found in the strains isolated in this study were extended-spectrum β-lactamases, followed by carbapenemases, being *bla*_CTXM_ and *bla*_SHV_ most prevalent in *E. coli*, *bla*_CTXM_ and *bla*_OXA-48_ in *K. pneumoniae* and *bla*_CTXM_ and AmpC hyperproduction in *Enterobacter* spp. Distribution of antibiotic resistance genes in *Enterobacteriaceae* strains isolated from transplant patients is shown in Table 1.

**Table 1:** Distribution of antibiotic resistance genes in *Enterobacteriaceae* strains.

	Mechanisms of resistance to extended-spectrum β-lactams and carbapenems										
Species (n° strains)	*bla*_CTX-M G1_ (90)	*bla*_CTX-M G8_ (1)	*bla*_CTX-M G9_ (43)	*bla*_TEM_ (3)	*bla*_SHV_ (18)	AmpC-hyperproducing (16)	*bla*_OXA-48_ (3)	*bla*_VIM_ (5)	*bla*_KPC_ (2)	*bla*_CTX-M G1 + _*bla*_OXA-48_ (27)	*bla*_CTX-M G9 + _*bla*_VIM_ (1)
*E. coli* (106)	50 (47.2%)	1 (0.9%)	33 (31.1%)	2 (1.9%)	15 (14.1%)	2 (1.9%)	—	—	—	2 (1.9%)	1 (0.9%)
*K. pneumoniae* (78)	39 (50.0%)	—	3 (3.8%)	1 (1.3%)	3 (3.8%)	—	2 (2.6%)	3 (3.8%)	2 (2.6%)	25 (32.0%)	—
*Enterobacter* spp. (25)	1 (4.0%)	—	7 (28.0%)	—	—	14 (56.0%)	1 (4.0%)	2 (8.0%)	—	—	—

Isolated from transplant patients.

### Hemagglutination in *E. coli* strains

Hemagglutination (HA) was observed in 23 strains (21.7%). HA was mannose sensitive (MS) in 9 strains (8.5%) and mannose resistant (MR) in 14 strains (13.2%) (Supplementary Table 1). We observed an inverse relationship between hemagglutinating strains and biofilm formation, because the isolates that show either sensitivity or resistance to the hemagglutination in presence of mannose are non- or weak biofilm former strains.

### Biofilm formation by *Enterobacteriaceae* strains

**Figure 1:**
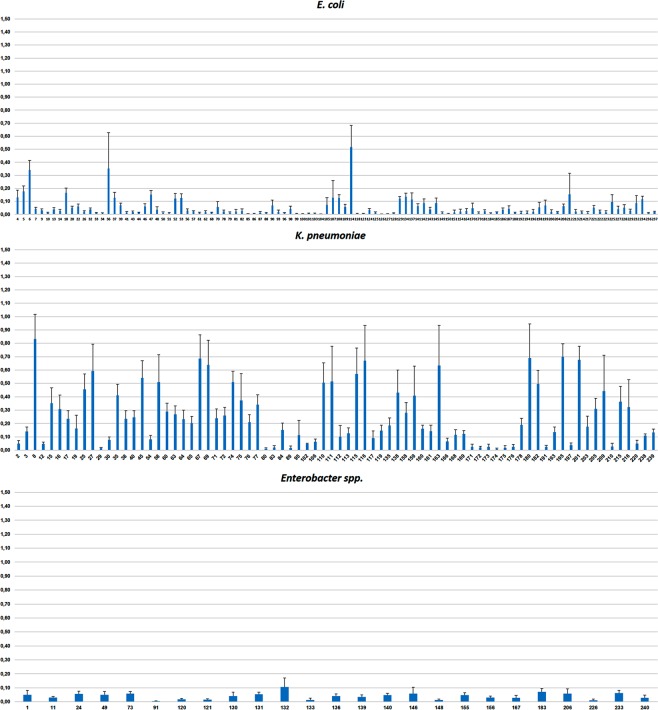
Quantification of biofilm formation was performed after Crystal Violet extraction and measurement (OD_620_). Values are presented as mean ± standard deviation of four independent experiments.

The ability of *Enterobacteriaceae* strains to form biofilms was quantified by crystal violet (CV) staining after 48 h at 37 °C. Bacterial growth was, in general, not strongly influenced by the culture conditions, as estimated by the colony forming units (CFU) of planktonic cells in 4 strains of each species, with numbers in the range from 1.95 × 10^8^ to 6.45 × 10^8^ CFUs/well. All the biofilms were found at the liquid-air interface. Overall, 16% of *E. coli* strains and 73% of *K. pneumoniae* strains showed moderate or strong biofilm production while only one *Enterobacter* strain showed moderate biofilm production (Fig. 1).

Seventy-two percent of 67 *K. pneumoniae* isolates shown moderate or strong biofilm formation (23 from renal transplants and 25 from hepatic transplants) whereas only 16 post-transplant *E. coli* isolates (19.5%) were biofilm forming strains, mostly from renal transplant recipients (n = 11).

**Figure 2:**
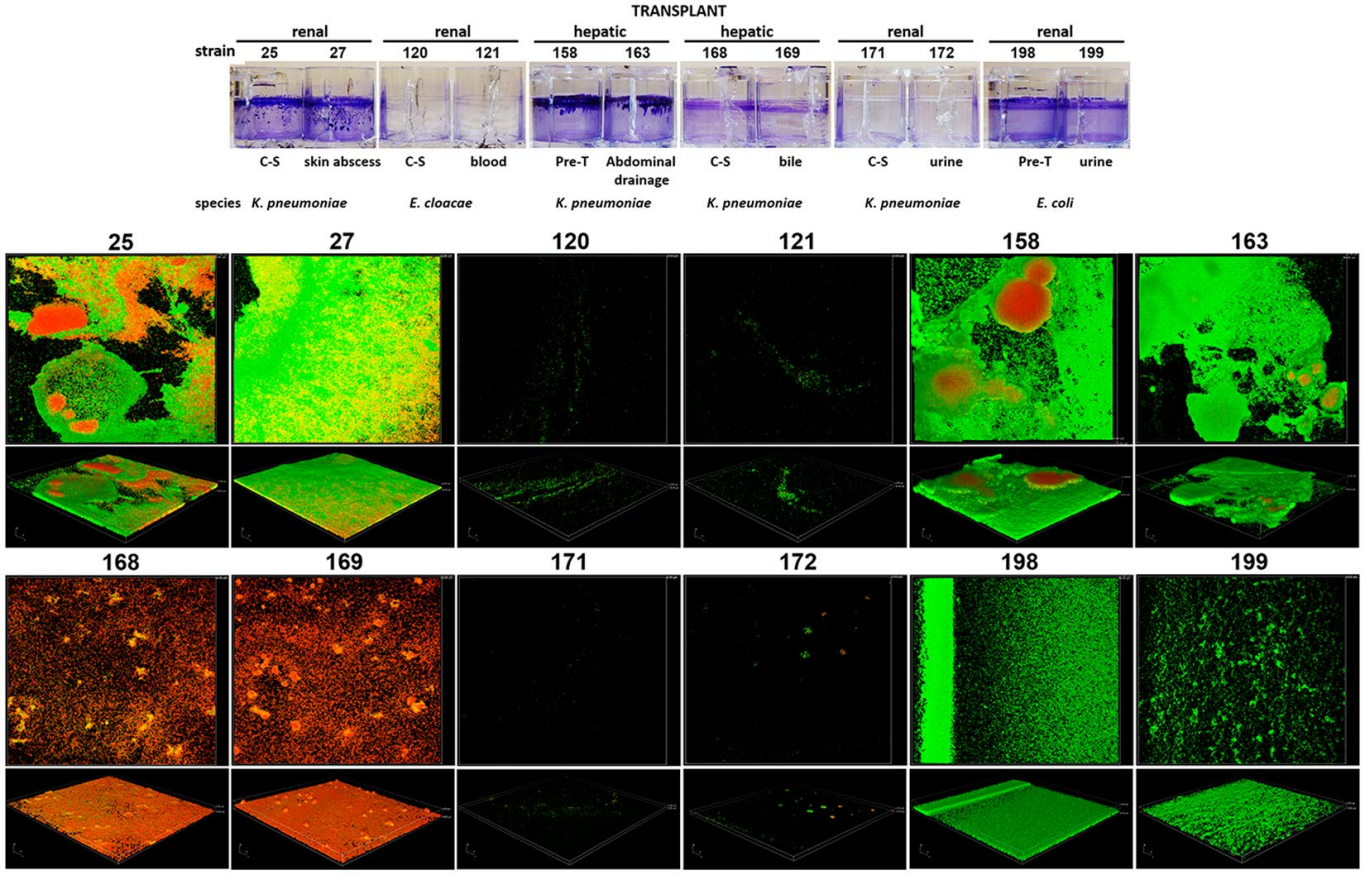
Biofilm formation by the six *Enterobacteriaceae* strains responsible of infections. These strains were compared with their respective isolates pre-transplant (Pre-T) or isolates from rectal swabs post-transplant (colonizing strains, C-S). Up: wells were stained with Crystal Violet inside 24-well plates. Source of isolation during infections is indicated. Down: representative examples of CLSM images of selected strains after biofilm formation. Bacteria were stained with the BacLight LIVE/DEAD viability kit. Live cells fluoresce in green with Syto 9 dye and dead cells are stained red with propidium iodide. Original magnification: ×200.

Morphology of biofilms was found to be variable, even for biofilms formed by the same species. Six representative infection-related isolates of the three species were selected for confocal microscopy analysis. These strains showed poor, weak, moderate/strong biofilm formation. All strains formed similar biofilms before and after re-emergence as infection related strains in transplant patients (Fig. 2). *K. pneumoniae* strains 25/27 and 158/163 formed biofilms with firmly packed, mushroom-shaped microcolony structures. Large red masses can be seen inside these structures, possibly due to the early formation of these microcolonies. These structures did not appear in the biofilms formed by *E. coli* (weak biofilm formation) which showed a thinner and less compact structure. The moderate biofilm producer *K. pneumoniae* 168/169 formed flat biofilms with loosely packed irregular microcolony structures and composed mainly by dead cells, as clearly showed by measurements of red/green pixel intensities inside biofilms (Supplementary Fig. 1). Moderate or poor biofilm producer strains (according to CV data) *K. pneumoniae* 171/172 and *Enterobacter* 120/121 respectively, showed few attached cells with loosely packed irregular microcolony structures with little heterogeneity.

### Infections by MDR in SOT recipients

Transplant recipients were colonized with MDR *Enterobacteriaceae* because the number of MDRE isolates post-transplant was 4.3 times the number of MDRE isolates pre-transplant (*E. coli* 81 vs. 25, *K. pneumoniae* 67 vs. 11, and *Enterobacter* 22 vs. 3).

**Figure 3:**
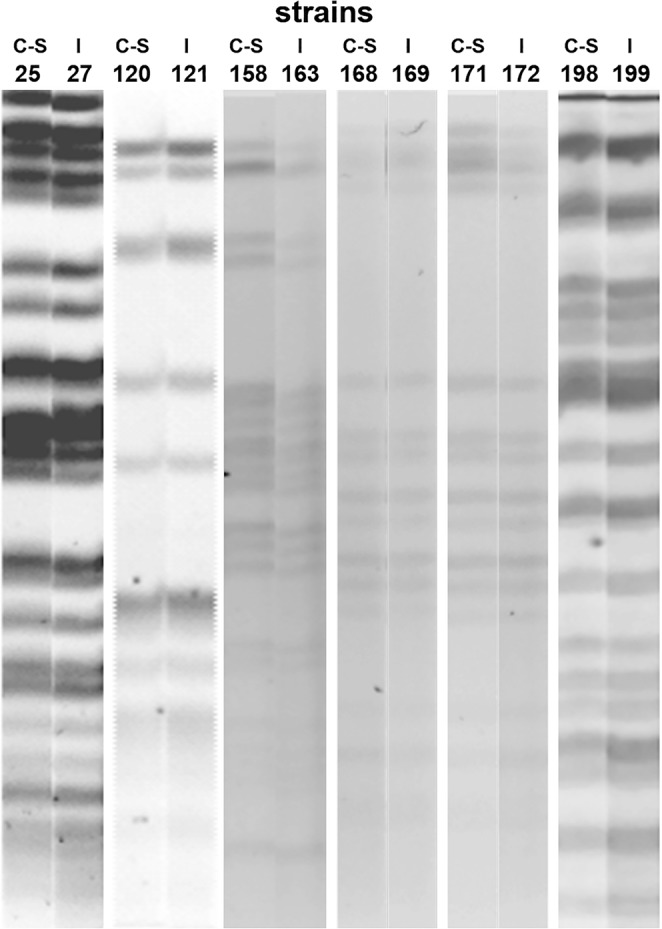
PFGE profiles of isolates from rectal swabs obtained pre-transplant or isolates from rectal swabs post-transplant (colonizing strains, C-S) and from infection-related strains (I).

Eight out of 158 (5.1%) patients enrolled in the project developed infection and six of the strains isolated from these patients (4%) were re-emergent strains detected in pre-transplant rectal swab samples or colonizers strains post-transplant (Fig. 3). All infection related strains were isolated during the first month post-transplant. These strains caused urinary infections (*K. pneumoniae* strain 172 and *E. coli* strain 199), bacteraemia (*Enterobacter cloacae* strain 121) and abdominal abscesses (*K. pneumoniae* strain 163) in renal transplant receptors, and skin abscess (*K. pneumoniae* strain 27) and cholecystitis (*K. pneumoniae* strain 169) in hepatic transplant receptors (Supplementary Table 1). Two other strains showing distinct pulsed-field gel electrophoresis (PFGE) patterns were not genetically related (not shown) and caused urinary tract infections (*E. coli* strain 228 and *K. pneumoniae* strain 239) in renal and hepatic transplant recipients, respectively. Patients infected with *K. pneumoniae* strains 27, 169 and 172 or *E. coli* strains 199 and 228 required additional antimicrobial treatment whereas transplant recipients infected with *E. cloacae* 121, and *K. pneumoniae* 163 and 239 did not. These infection related strains from hepatic transplant recipients formed moderate or strong biofilms, whereas strains from renal transplant recipients produced the full range of biofilms (from weak to strong biofilm production). Data on antibiotic resistance, biofilm formation and HA for the eight strains responsible for infection of transplant recipients are summarized in Table 2.

**Table 2:** Characteristics of the eight strains responsible for infection of transplant recipients.

Strain	post-transplant infection	Transplant	Species	^a^Antibiotic resistance	Resistance genes	^b^HA	^c^Biofilm
25	—	Renal	*K. pneumoniae*	AMX, AMC, AZT, CAZ, CIP, CTX, FEP, FOS, GN, NET, PIP, SXT, TO, TZP	*bla*CTXM-group1	—	SB
27	**Skin abscess**	Renal	*K. pneumoniae*	AMX, AMC, AZT, CAZ, CTX, FEP, GN, NET, PIP, SXT, TGC, TO, TZP	*bla*CTXM-group1	—	SB
120	—	Renal	*E. cloacae*	AMX, AMC, CTX, FOS, FOX, NAL, PIP, TGC, TZP	*bla*CTXM-group9	—	NB
121	**Blood**	Renal	*E. cloacae*	AMX, AMC, CTX, FOS, FOX, NAL, PIP, TGC, TZP	*bla*CTXM-group9	—	NB
158	—	Hepatic	*K. pneumoniae*	AMX, AMC, AZT, CAZ, CIP CTX, LEV, NAL, PIP, SXT	*bla*CTXM-group1	—	MB
163	**Abdominal drainage**	Hepatic	*K. pneumoniae*	AMX, AMC, AZT, CAZ, CIP CTX, FEP, FOX, LEV, NAL, PIP, SXT, TGC	*bla*CTXM-group1	—	SB
168	—	Hepatic	*K. pneumoniae*	AMX, AMC, AZT, CAZ, CIP, COL, CTX, ETP, FEP, FOS, FOX, IMP, LEV, MRP, NAL, NET, PIP, TO, TZP	*bla*CTXM-group1 + *bla*OXA-48	—	MB
169	**Bile**	Hepatic	*K. pneumoniae*	AMX, AMC, AZT, CAZ, CIP, CTX, ETP, FEP, FOS, FOX, IMP, LEV, MRP, NAL, PIP, TGC, TO, TZP	*bla*CTXM-group1 + *bla*OXA-48	—	MB
171	—	Renal	*K. pneumoniae*	AMX, AMC, AZT, CTX, CAZ, CIP, ETP, FEP, FOS, FOX, GN, LEV, NAL, NET, PIP, TO, TGC, TZP	*bla*CTXM-group1 + *bla*OXA-48	—	NB
172	**Urine**	Renal	*K. psneumoniae*	AMX, AMC, AZT, CTX, CAZ, CIP, ETP, FEP, FOS, FOX, LEV, NAL, PIP, SXT, TGC, TZP	*bla*CTXM-group1 + *bla*OXA-48	—	NB
198	—	Renal	*E. coli*	AMX, AMC, AZT, CTX, CIP, LEV, NAL, NET, PIP, SXT, TO	*bla*CTXM-group1	MRHA	WB
199	**Urine**	Renal	*E. coli*	AMX, AMC, AZT, CTX, CIP, LEV, NAL, NET, PIP, SXT, TO	*bla*CTXM-group1	MRHA	WB

^a^AMX, amoxicillin; AMC, amoxicillin-clavulanic acid; AMK; amikacin; AZT, aztreonam; CAZ, ceftazidime; CIP, ciprofloxacin; COL, colistin; CTX, cefotaxime; ERT, ertapenem; FEP, cefepime; FOS, fosfomycin; FOX, cefoxitin; GN, gentamicin; IMP, imipenem; LEV, levofloxacin; MRP, meropenem; NAL, nalidixic acid; NET, netilmicin; PIP, piperacillin; SXT, trimethoprim–sulfamethoxazole;TIG, tigecycline TO, tobramycin; TZP, piperacillin-tazobactam. ^b^MRHA, mannose-resistant hemagglutination. ^c^Biofilm formation: NB, non-biofilm; WB, weak; MB, moderate; SB, strong.

## Discussion

Postoperative care of transplant recipients implies frequent manipulations, the use of invasive medical devices, and the frequent use of extensive antibiotic therapy, which further contributes to the selection of multidrug-resistant bacteria. This cycle is difficult to avoid and leads to a scenario with narrowed therapeutic options. Moreover, patients awaiting transplantation could also become colonized with these bacteria.

Biofilm formation is one important characteristic of *E. coli* and other *Enterobacteriaceae*^27^. Biofilm producing bacteria may be responsible for many nosocomial infections. Biofilm characteristics protect bacteria from the host immune system along with the antibiotics, therefore, the role of biofilms during bacterial infections now constitutes an active field of research.

In this study, the ability of biofilm formation *in vitro* varied extensively among the *E. coli* isolates, and was independent of the type of transplant. The percentage of strains of *K. pneumoniae* producing biofilm was much higher than in *E. coli* or *Enterobacter* strains and was similar in kidney and liver transplants. In fact, the rate of biofilm formation by *Enterobacter* strains was very low (4%). Other studies showed similar or slightly higher proportion of *Enterobacter* strains that can form biofilms^28,29^. The high rate of biofilm-producing *K. pneumoniae* and the variability among *E. coli* isolates found in this study are in concordance with other studies evaluating MDR strains^14,30,31^.

We evaluated the possible relationship between biofilm formation and the presence of antimicrobial resistance genes. No relationship was found between strong biofilm formation and the presence of resistance genes in the three bacterial groups; but among the non-biofilm producers of *K. pneumoniae* (OD_620_ ≤ 0.05, n = 16) 11 strains were positive for the combination of CTXM-G1 + OXA-48 and other 3 carried the Verona integrin-encoded metallo-β-lactamase (VIM). Only 3 *K. pneumoniae* strains which were unable to form biofilms carried out other different resistant genes (strains 80, 83, and 102).

Looking at the type of transplant, there is no significant difference between strains isolated in liver transplantation with respect to kidney transplantation (unpaired t-test, *p* > 0.05).

*K. pneumoniae*, *E. coli* and *Enterobacter* strains isolated from rectal swabs prior to transplant or isolated from rectal swabs during the first weeks after transplantation were found to cause infections in 6 patients. These infection-related strains showed the same PFGE pattern and formed similar biofilms that their homologous colonizing strains. However, two *K. pneumoniae* strains (27 and 163) formed more biofilm that their corresponding pre-transplant isolates (25 and 158, paired t-test: *p* = 0.008 and *p* = 0.014 respectively). Although infections can occur at any time after transplantation, their incidence is highest in the first post-operative month^32–34^. In our study, these reemergent strains cause infections during the first month post-transplant, indicating that early detection and decolonization of these MDR on time is of critical importance to avoid further complications. However, enhanced surveillance monitoring for longer periods regarding opportunistic infections must also be addressed.

HA in *E. coli* and other *Enterobacteriaceae* species is mediated by fimbriae. Whereas in chronic conditions like urolithiasis biofilm plays an important role in persistence of infection, hemagglutination mediated by type I fimbriae, which bind to a mannose-containing receptor, are found in most urinary isolates where plays an important role in acute urinary tract infections^31,35^. The expression of type I fimbriae is indicated by MS hemagglutination while MR hemagglutination can be mediated by several types, such as P-fimbriae and DR fimbriae. Thus, MRHA-positive isolates can be considered most likely having P or DR fimbriae instead of type I fimbriae. Several authors have found a positive correlation between biofilm formation and the presence of type I pili in *Enterobacteriaceae* strains, including *E. coli*^36,37^. In this study, the number of *E. coli* strains that showed MRHA (n = 14) was higher than those that showed MSHA (n = 9). Interestingly, the 2 *E. coli* strains (199 and 228) recovered from infections were both MRHA and produced a very fast hemagglutination of human red blood cells. Of note, *E. coli* hemagglutinating strains but these two, were all poor biofilm producers; therefore, there was no correlation between MR or MS hemagglutination of human erythrocytes and biofilm formation.

In this report, we have demonstrated that *E. coli* and *K. pneumoniae* isolates display a high degree of phenotypic variability, being *K. pneumoniae* strains the greatest biofilm producers, and that *Enterobacter* isolates not form even moderate biofilms *in vitro*. Investigation of the mechanisms responsible for strong biofilm formation in *K. pneumoniae* is needed and would clarify some pathogenicity attributes of these bacteria in transplant recipients. On the other hand, as some isolates of the three genera analysed in this study cause infections but did not produce biofilms *in vitro*, it would be worthwhile to identify the presence and significance of other virulence factors responsible for the development of recurrent infections after SOT.

## Methods

### Study population and setting

The present prospective cohort study was conducted between August 2014 and April 2018 in seven University Hospitals from five Spanish regions. This national project (ENTHERE study) focused on the study of intestinal colonization and infections with MDRE in patients with kidney, liver, and kidney/pancreas transplants. Transplant patients who have been treated with antibiotics active against MDR *Enterobacteriaceae* (in the 30 days prior to inclusion in the study) such us colistin, carbapenems, amikacin or tigecycline, or patients who are participating in another study or clinical trial that includes active antibiotic treatment were not included in this study.

### Bacterial strains

Over a period of 33 months, from October 2014 to June 2017, a total of 209 MDRE isolates defined as AmpC-hyperproducing and/or extended-spectrum β-lactamases (ESBLs) or carbapenemases producers, were included in this study. MDRE were obtained from 158 patients with kidney (n = 93) or liver transplant (n = 60) or both kidney and pancreatic transplant (n = 5) from the ENTHERE study. Among these, 106 were *Escherichia coli*, 78 were *Klebsiella pneumoniae*, and 25 were *Enterobacter* spp. (Supplementary Table 1). Bacteria were isolated from rectal swabs (in the 48 h previous to transplant and weekly samples till 4–6 weeks after transplantation) and directly inoculated onto chromID® ESBL and chromID® CARBA chromogenic agar plates (bioMérieux, Marcy L’Etoile, France). Colonies were subcultured on blood agar and MacConkey agar and preliminary identification and antimicrobial susceptibility testing the Vitek 2 compact system (bioMérieux, France) was used. Identification was confirmed by MALDI-TOF using the Vitek MS (bioMérieux) system, in accordance with manufacturer’s instructions. Stock cultures were frozen at −80 °C with 20% (vol/vol) glycerol.

### Molecular characterization of resistance genes

Standard PCR were used to amplify several genes encoding extended-spectrum β-lactamases (*bla*_TEM_, *bla*_SHV_ and *bla*_CTXM_) and carbapenemases (*bla*_KPC_, *bla*_VIM_, *bla*_IMP_, *bla*_NDM_ and *bla*_OXA-48_). PCR multiplex plasmid-mediated AmpC (*bla*_CIT_, *bla*_FOX_, *bla*_MOX_, *bla*_DHA_, *bla*_ACC_ and *bla*_EBC_) was performed as described elsewhere^38^.

Genomic DNA was extracted using an InstaGene^TM^ Matrix Kit (Bio-Rad, Madrid, Spain) according to the manufacturer’s instructions. Then, 1 µL was added to a reaction mixture containing 1× KAPA Taq ReadyMix (KapaBiosystems, Wilmington, USA) and 0.5 µM of each primer. Amplification conditions were 94 °C for 5 min, followed by 30 cycles of 94 °C for 30 s, primer annealing at 55, 60 or 64 °C for 30 s and 72 °C for 1 min, and a final elongation at 72 °C for 7 min. PCR products were analysed on 2% (wt/vol) agarose gels stained with ethidium bromide 10 µg/mL and visualized by UV transillumination.

The amplified products were purified with a QIAquick PCR Purification Kit (QIAGEN Inc., Barcelona, Spain). DNA sequences on both strands were determined using an external resource (Macrogen Inc., Amsterdam, The Netherlands). Primers used in the detection of antimicrobial resistance genes, expected amplicon sizes, annealing temperatures, and references are shown in Supplementary Table 2.

### Molecular fingerprinting

Repetitive extragenic palindromic PCR (REP-PCR) typing was performed on all isolates as described elsewhere^39^. Amplicons were run in a 1.5% agarose gel for 100 min, stained with ethidium bromide, and photographed. When at least two different bands were observed among isolates clonal relationship was also determined by PFGE. For this purpose, bacterial DNA embedded in agarose plugs was digested with the restriction enzyme *XbaI* and DNA separation was then performed in a CHEF-DRIII variable angle system (Bio-Rad, California, USA). Finally, the PFGE patterns were analysed with Fingerprinting II v4.5 software (Bio-Rad) and interpreted according to the criteria established by Tenover *et al*.^40^.

### Biofilm formation

Biofilm formation was estimated in 24-well polystyrene plates (Corning, Costar) as previously described^41^. Briefly, *Enterobacteriaceae* strains were grown in Luria broth medium for 24 h at 37 °C with shaking, and a 1:1,000 dilution was prepared in Luria broth. Twenty-five microliters were placed in each well containing 1.5 ml of culture medium. The plates were incubated for 48 h at 37 °C in static. Planktonic cells were removed and wells containing biofilms were rinsed three times with distilled water and the remaining adherent bacteria were stained with 2 ml/well of crystal violet (0.7% [wt/vol] solution; Sigma-Aldrich) for 12 min. Excess stain was removed by washes with distilled water. CV was extracted by acetic acid (33% [vol/vol]), and the plates were incubated at room temperature in a bench orbital shaker for 1 min at 400 rpm to release the dye into the solution. Then, two samples of 100 µl from each well were transferred to a 96-well flat-bottom plate, and the amount of dye was determined at 620 nm using a microplate reader (Multiskan FC; Thermo Fisher). In each experiment, results were corrected for background staining by subtracting the value for crystal violet bound to uninoculated controls. The assays were performed 4 times for each isolate and the mean ± SD was reported. Strains were classified as non-adherent, weak, moderate or strong biofilm producers using the following criteria: OD ≤ 0.05, non-biofilm producer; OD > 0.05–0.1 weak biofilm producer; OD > 0.1–0.3 moderate biofilm producer; and OD > 0.3 strong biofilm producer.

### Confocal laser scanning microscopy (CLSM)

Biofilm architecture of selected strains was studied in 4-well µ-slides (Ibidi, Martinsried, Germany) as previously described^41^. Briefly, the slides were placed inclined (~45°) into an incubator to form a liquid-air interface and after 48 h at 37 °C, unfixed planktonic cells were removed by rinsing with saline (0.85% NaCl), and bacterial viability within biofilms was determined by using the BacLight LIVE/DEAD bacterial viability kit (Molecular Probes Inc.). A series of optical sections was obtained with a Nikon A1R confocal microscope; the excitation wavelengths were 488 nm (green) and 561 nm (red), and 500 to 550 nm and 570 to 620 nm emission filters were used, respectively. Images at the liquid-air interface were captured at random with a 20× Plan Apo (numerical aperture [NA], 0.75) objective. Reconstructions of confocal sections and quantitative measurements were performed using NIS-Elements software, version 3.2.

### Hemagglutination and HA inhibition tests

To identify other factors associated with biofilm formation in *E. coli* strains, we studied their hemagglutinating activities with human group A erythrocytes and the mannose sensitivities of these agglutinations as previously described^41^. Human erythrocytes were obtained from healthy volunteers after informed consent. Hemagglutination tests were performed on microscope slides using 10% suspensions of human group A erythrocytes. *E. coli* were cultured at 37 °C for 24 h in Luria medium, washed, and suspended in PBS to a concentration of ~5 × 10^9^ CFUs per ml. Twenty-five microliters of cultures were mixed with 50 µl of erythrocytes with or without 1% D-mannose (Sigma). Agglutination of erythrocytes was examined visually after a short period (up to 1 min) of rocking at RT. Hemagglutination was considered resistant to mannose when it occurred despite the presence of mannose and sensitive to mannose when it was inhibited by the presence of this carbohydrate.

### Statistical analysis

Data were described with means and standard deviation. Comparisons of the quantitative data was carried out by comparing means with the paired or unpaired t-test as corresponding. The alpha error was set at 0.05, and all *p* values were bilateral.

### Ethical approval and informed consent

The study was performed in accordance with the Declaration of Helsinki. The protocol was reviewed and approved by the Institutional Ethics Committee at all seven participating hospitals according to local standards (EUDRAT-). The participating hospitals were: Hospital Universitario Marqués de Valdecilla (Santander), Coordinating Center; Hospital Universitario Cruces (Baracaldo); Hospital Clínic Universitari (Barcelona); Hospital General Universitario Gregorio Marañón (Madrid); Hospital Universitario 12 de Octubre (Madrid); Hospital Universitario Reina Sofía (Córdoba) and Hospital Universitario Ramón y Cajal (Madrid). Informed consent was obtained from each patient.

## Supplementary information

Dataset 1

## Data Availability

All data generated or analysed during this study are included in this published article (and its Supplementary Information files).
