# Author Correction: Biofilm formation by multidrug resistant *Enterobacteriaceae* strains isolated from solid organ transplant recipients

**DOI:** 10.1038/s41598-020-60496-3

**Published:** 2020-04-29

**Authors:** José Ramos-Vivas, Itziar Chapartegui-González, Marta Fernández-Martínez, Claudia González-Rico, Jesús Fortún, Rosa Escudero, Francesc Marco, Laura Linares, Miguel Montejo, Maitane Aranzamendi, Patricia Muñoz, Maricela Valerio, Jose María Aguado, Elena Resino, Irene Gracia Ahufinger, Aurora Paz Vega, Luis Martínez-Martínez, María Carmen Fariñas, The ENTHERE Study Group, the Group for Study of Infection in Transplantation of the Spanish Society of Infectious Diseases and Clinical Microbiology (GESITRA-SEIMC) and the Spanish Network for Research in Infectious Diseases (REIPI), Juan Carlos Ruiz San Millán, Emilio Rodrigo, Fernando Casafont Morencos, Emilio Fabrega, Antonio Cuadrado, Concepción Fariñas-Alvarez, Mónica Gozalo, Francisco Arnaíz de las Revillas, Pilar Martín Dávila, Adolfo Martínez, Patricia Ruíz Garbajosa, Asunción Moreno, Marta Bodro, María Fernanda Solano, María José Blanco, Javier Nieto, Marina Machado, María Olmedo, Sara Rodríguez Fernández, Cristina Rincón Sanz, Teresa Vicente Rangel, Caroline Agnelli Bento, Alicia Galar Recalde, Alia Eworo, Fernando Anaya Fernández-Lomana, María Luisa Rodríguez-Ferrero, Luis Alberto Sánchez Cámara, Fernando Chaves, Julián de la Torre Cisneros

**Affiliations:** 1 Instituto de Investigación Valdecilla-IDIVAL, Avd. Cardenal Herrera Oria, 39011 Santander, Spain; 2 Service of Microbiology, Hospital Universitario Marqués de Valdecilla, Avd. Valdecilla, 39008 Santander, Spain; 3 Infectious Diseases Unit. Hospital Universitario Marqués de Valdecilla, Santander, Spain. Avd. Valdecilla, 39008 Santander, Spain; 4 Infectious Diseases Department, Hospital Universitario Ramón y Cajal, Ctra. Colmenar Viejo, km. 9, 100, 28034 Madrid, Spain; 5 Service of Microbiology, Hospital Clínic-IDIBAPS, Universidad de Barcelona, Carrer de Villarroel, 170, 08036 Barcelona, Spain; 6 Infectious Diseases Service, Hospital Clínic-IDIBAPS, Universidad de Barcelona, Carrer de Villarroel, 170, 08036 Barcelona, Spain; 7 Infectious Diseases Unit, Hospital Universitario Cruces, Plaza de Cruces, S/N, 48903 Baracaldo, Vizcaya Spain; 8 Service of Microbiology, Hospital Universitario Cruces, Plaza de Cruces, S/N, 48903 Baracaldo, Vizcaya Spain; 9 Clinical Microbiology and Infectious Diseases, Hospital General Universitario Gregorio Marañón, Calle del Dr. Esquerdo, 46, 28007 Madrid, Spain; 10 Infectious Diseases Unit, Hospital Universitario 12 de Octubre, Av. Córdoba, s/n, 28004 Madrid, Spain; 11 Service of Microbiology, Hospital Universitario Reina Sofía, Av. Menéndez Pidal, s/n, 14004 Córdoba, Spain; 12 Infectious Diseases Unit, Hospital Universitario Reina Sofía, Av. Menéndez Pidal, s/n, 14004 Córdoba, Spain; 13 Nephrology Department, Hospital Universitario Marqués de Valdecilla, Santander, Spain; 14 Liver Unit, Hospital Universitario Marqués de Valdecilla, Santander, Spain; 15 Quality Unit, Hospital Universitario Marqués de Valdecilla, Santander, Spain; 16 Transplant Coordination, Hospital Universitario Ramón y Cajal, Madrid, Spain; 17 Service of Microbiology, Hospital Universitario Ramón y Cajal, Madrid, Spain; 18 Department of Nephrology, Hospital General Universitario Gregorio Marañón, Madrid, Spain; 19 Service of Microbiology, Hospital Universitario 12 de Octubre, Madrid, Spain

Correction to: *Scientific Reports* 10.1038/s41598-019-45060-y, published online 20 June 2019

The original version of this Article contained a typographical error in the spelling of the author Luis Martínez-Martínez, which was incorrectly given as Luis Martínez.

In addition, this Article contained a typographical error in the following affiliation.

Infectiuos Diseases Unit, Hospital Universitario Cruces, Plaza de Cruces, S/N, 48903, Baracaldo, Vizcaya,Spain

now reads:

Infectious Diseases Unit, Hospital Universitario Cruces, Plaza de Cruces, S/N, 48903, Baracaldo, Vizcaya, Spain

These errors have now been corrected in the PDF and HTML versions of the Article, and in the accompanying Supplementary Material File.

