# Supplementary material for: Biofilm formation by multidrug resistant Enterobacteriaceae strains isolated from solid organ transplant recipients
Source: Sci Rep. 2019 Jun 20;9:8928. doi: 10.1038/s41598-019-45060-y (PMC6586660; doi:10.1038/s41598-019-45060-y)
Supplement: Supplementary file 1 — Dataset 1 [file 41598_2019_45060_MOESM1_ESM.pdf]

Biofilm formation by multidrug resistant *Enterobacteriaceae* strains isolated from solid organ transplant recipients

José Ramos-Vivas[1], Itziar Chapartegui-González[1], Marta Fernández-Martínez[2], Claudia González-Rico[3], Jesús Fortún[4], Rosa Escudero[4], Francesc Marco[5], Laura Linares[6], Miguel Montejo[7], Maitane Aranzamendi[8], Patricia Muñoz[9], Maricela Valerio[9], Jose María Aguado[10], Elena Resino[10], Irene Gracia Ahufinger[11], Aurora Paz Vega[12], Luis Martínez-Martínez[2,11], María Carmen Fariñas[1,3], *for the ENTHERE Study Group, the Group for Study of Infection in Transplantation of the Spanish Society of Infectious Diseases and Clinical Microbiology (GESITRA-SEIMC) and the Spanish Network for Research in Infectious Diseases (REIPI).

[1]José Ramos-Vivas, Instituto de Investigación Valdecilla-IDIVAL, Avd. Cardenal Herrera Oria, 39011 Santander, Spain. e-mail: jvivas@idival.org

[1]Itziar Chapartegui-González, Instituto de Investigación Valdecilla-IDIVAL, Avd. Cardenal Herrera Oria, 39011 Santander, Spain. e-mail: ichapartegui@idival.org

[2]Marta Fernández-Martínez, Service of Microbiology, Hospital Universitario Marqués de Valdecilla, Avd. Valdecilla, 39008 Santander, Spain. e-mail: mfmtorrelavega@yahoo.es

[3]Claudia González-Rico, Infectious Diseases Unit. Hospital Universitario Marqués de Valdecilla, Santander, Spain. Avd. Valdecilla, 39008 Santander, Spain. e-mail: claugonzalez@humv.es

[4]Jesús Fortún, Infectious Diseases Department, Hospital Universitario Ramón y Cajal, Ctra. Colmenar Viejo, km. 9, 100, 28034 Madrid, Spain. e-mail: fortunabete@gmail.com

[4]Rosa Escudero, Infectious Diseases Department, Hospital Universitario Ramón y Cajal, Ctra. Colmenar Viejo km. 9, 100, 28034 Madrid, Spain. e-mail: rosa.escudero0@gmail.com

[5]Francesc Marco, Service of Microbiology, Hospital Clínic-IDIBAPS, Universidad de Barcelona, Carrer de Villarroel, 170, 08036 Barcelona, Spain. e-mail: fmarco@clinic.ub.es

[6]Laura Linares, Infectious Diseases Service, Hospital Clínic-IDIBAPS, Universidad de Barcelona, Carrer de Villarroel, 170, 08036 Barcelona, Spain. e-mail: laura.linares.gonzalez@gmail.com

[7]Miguel Montejo, Infectious Diseases Unit, Hospital Universitario de Cruces, Plaza de Cruces, S/N, 48903 Baracaldo, Vizcaya Spain. e-mail: JOSEMIGUEL.MONTEJOBARANDA@osakidetza.eus

[8]Maitane Aranzamendi, Service of Microbiology, Hospital Universitario de Cruces, Plaza de Cruces, S/N, 48903 Baracaldo, Vizcaya, Spain. e-mail: maitanearanzamendi@yahoo.es

[9]Patricia Muñoz, Clinical Microbiology and Infectious Diseases, Hospital General Universitario Gregorio Marañón, Calle del Dr. Esquerdo, 46, 28007 Madrid, Spain. e-mail: pmunoz@micro.hggm.es

[9]Maricela Valerio, Clinical Microbiology and Infectious Diseases, Hospital General Universitario Gregorio Marañón, Calle del Dr. Esquerdo, 46, 28007 Madrid, Spain. e-mail: mavami_valerio@yahoo.com.mx

[10]José María Aguado, Infectious Diseases Unit, Hospital Universitario 12 de Octubre, Avd. Córdoba, s/n 28004 Madrid, Spain. e-mail: jaguadog1@gmail.com

[10]Elena Resino, Infectious Diseases Unit, Hospital Universitario 12 de Octubre, Avd. Córdoba, s/n 28004 Madrid, Spain. e-mail: elenaresinofoz@gmail.com

[11]Irene Gracia Ahufinger, Service of Microbiology, Hospital Universitario Reina Sofía, Avd. Menéndez Pidal, s/n, 14004 Córdoba, Spain. e-mail: irene.gracia.sspa@juntadeandalucia.es

[12]Aurora Paz Vega, Infectious Diseases Unit, Hospital Universitario Reina Sofía, Avd. Menéndez Pidal, s/n, 14004 Córdoba, Spain. e-mail: aumapave@hotmail.com

[2,11]Luis Martínez-Martínez, Service of Microbiology. Current address: Hospital Universitario Reina Sofía, Avd. Menéndez Pidal, s/n, 14004 Córdoba, Spain. e-mail: luis.martinez.martinez.sspa@juntadeandalucia.es

[1,3]María Carmen Fariñas, Infectious Diseases Unit. Hospital Universitario Marqués de Valdecilla, University of Cantabria. Avd. Valdecilla s/n 39008 Santander, Spain. e-mail: mcarmen.farinas@scsalud.es

Correspondence: María Carmen Fariñas**,** Infectious Diseases Unit. Hospital Universitario Marqués de Valdecilla, University of Cantabria. Avd. Valdecilla s/n 39008 Santander. e-mail address: mcarmen.farinas@scsalud.es

Phone: +34-942 202520, Fax: +34-942 202750

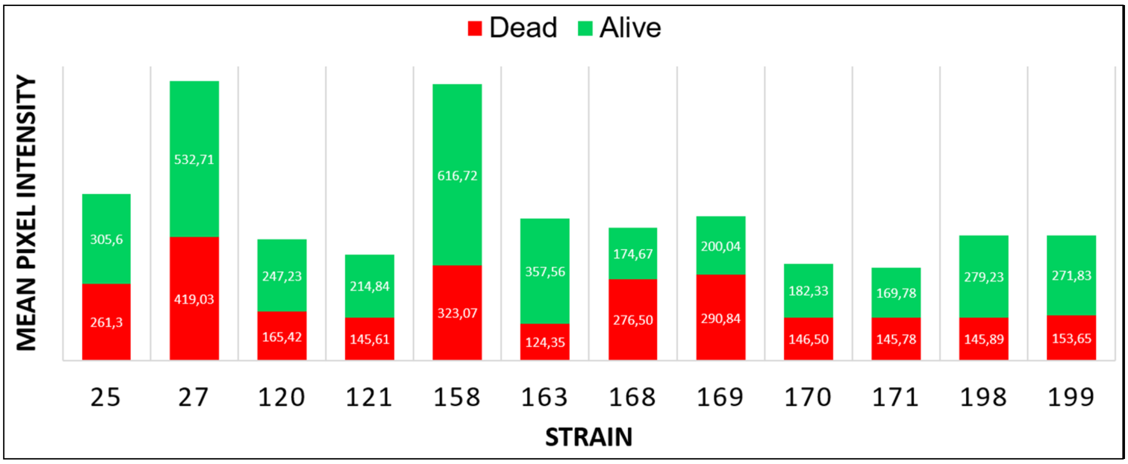

Supplementary Figure 1.

Confocal Laser Scanning Microscopy of live/dead cells.

Bacteria inside biofilms were stained with the BacLight LIVE/DEAD viability kit. Graph shows the average of pixel intensity measurements of live and dead cells of three representative random biofilms formed by the six strains isolated before and after transplantation.

Supplementary Table S1

nº strain	[a]Strain code	HOSPITAL	[b]date isolation pre/postransplant (week) or post-transplant infection	Transplant	*Species*	Resistance genes	[c]HA	[d]Biofilm
1	HU12O-01	12OCTUBRE	3	Renal	*E. cloacae*	VIM	-	WB
2	HU12O-02	12OCTUBRE	2	Renal	*K. pneumoniae*	CTXM-G1 + OXA-48	-	NB
3	HU12O-03	12OCTUBRE	2	Renal	*K. pneumoniae*	CTXM-G1	-	MB
4	HU12O-06	12OCTUBRE	2	Renal	*E. coli*	CTXM-G9	-	MB
5	HU12O-07	12OCTUBRE	3	Renal	*E. coli*	CTXM-G9	-	MB
6	HU12O-09	12OCTUBRE	1	Renal	*E. coli*	CTXM-G1	MSHA	SB
7	HU12O-11	12OCTUBRE	1	Renal	*E. coli*	CTXM-G1	-	NB
8	HU12O-13	12OCTUBRE	2	Renal	*K. pneumoniae*	CTXM-G1	-	SB
9	HU12O-18	12OCTUBRE	2	Renal	*E. coli*	CTXM-G9	-	NB
10	HU12O-20	12OCTUBRE	2	Renal	*E. coli*	CTXM-G1	-	NB
11	HCl-02	CLINIC	1	Renal	*E. cloacae*	Hiper-AmpC-c	-	NB
12	HCl-08	CLINIC	3	Renal	*K. pneumoniae*	VIM	-	NB
13	HCl-01	CLINIC	Pre-T	Renal	*E. coli*	CTXM-G1	-	NB
14	HCl-03	CLINIC	1	Renal	*E. coli*	CTXM-G1	-	NB
15	HCl-09	CLINIC	3	Renal	*K. pneumoniae*	CTXM-G1	-	SB
16	HCl-12	CLINIC	2	Hepatic	*K. pneumoniae*	CTXM-G1	-	SB
17	HCl-29	CLINIC	5	Hepatic	*K. pneumoniae*	CTXM-G1	-	MB
18	HCl-11	CLINIC	Pre-T	Renal	*E. coli*	CTXM-G9	-	MB
19	HCl-19	CLINIC	3	Renal	*K. pneumoniae*	CTXM-G1	-	MB
20	HCl-15	CLINIC	1	Hepatic	*E. coli*	CTXM-G9	-	WB
22	HCl-22	CLINIC	3	Hepatic	*E. coli*	CTXM-G9+ VIM	-	WB
24	HCl-33	CLINIC	3	Renal	*E. cloacae*	CTXM-G9	-	WB
25	HCl-20	CLINIC	1	Renal	*K. pneumoniae*	CTXM-G1	-	SB
26	HCl-30	CLINIC	3	Renal	*E. coli*	SHV	MSHA	NB
27	HCl-47	CLINIC	**Skin abscess**	Renal	*K. pneumoniae*	CTXM-G1	-	SB
29	HCl-35	CLINIC	2	Renal	*K. pneumoniae*	CTXM-G1 + OXA-48	-	NB
30	HCl-36	CLINIC	2	Renal	*K. pneumoniae*	CTXM-G1	-	WB
32	HCl-43	CLINIC	Pre-T	Renal	*E. coli*	SHV	-	NB
33	HCl-44	CLINIC	Pre-T	Renal	*E. coli*	CTXM-G1	-	NB
34	HCl-32	CLINIC	3	Hepatic	*E. coli*	CTXM-G1	-	NB
35	HCl-48	CLINIC	4	Renal	*K. pneumoniae*	CTXM-G1	-	SB
36	HCl-55	CLINIC	4	Hepatic	*E. coli*	CTXM-G9	-	SB
37	HCl-57	CLINIC	3	Renal	*E. coli*	CTXM-G9	-	MB
38	HCl-59	CLINIC	2	Hepatic	*K. pneumoniae*	CTXM-G1	-	MB

39	HCl-60	CLINIC	2	Hepatic	*E. coli*	CTXM-G1	-	WB
40	HCL-64	CLINIC	4	Renal	*K. pneumoniae*	CTXM-G1	-	MB
41	HCL-65	CLINIC	2	Renal	*E. coli*	CTXM-G9	MRHA	NB
43	HCL-68	CLINIC	1	Hepatic	*E. coli*	SHV	-	NB
44	HCl-72	CLINIC	1	Renal	*E. coli*	CTXM-G9	-	NB
45	HCl-73	CLINIC	2	Hepatic	*K. pneumoniae*	CTXM-G1	-	SB
46	HCl-74	CLINIC	5	Hepatic	*E. coli*	CTXM-G1	-	WB
47	HCl-77	CLINIC	5	Renal	*E. coli*	CTXM-G1	-	MB
48	HCl-78	CLINIC	2	Hepatic	*E. coli*	CTXM-G1	MRHA	NB
49	HCl-79	CLINIC	2	Hepatic	*E. aerogenes*	Hiper-AmpC-c	-	WB
50	HCl-82	CLINIC	3	Renal	*E. coli*	CTXM-G1	-	NB
51	HCl-83	CLINIC	4	Renal	*E. coli*	CTXM-G1	-	NB
52	HCl-85	CLINIC	2	Renal	*E. coli*	CTXM-G9	-	MB
53	HCl-87	CLINIC	3	Renal	*E. coli*	CTXM-G9	-	MB
54	HCl-88	CLINIC	2	Hepatic	*K. pneumoniae*	CTXM-G9	-	WB
56	HCl-93	CLINIC	Pre-T	Renal	*E. coli*	CTXM-G1	MRHA	NB
57	HCl-94	CLINIC	1	Renal	*E. coli*	CTXM-G1	MRHA	NB
58	HCl-95	CLINIC	1	Renal	*K. pneumoniae*	CTXM-G1	-	SB
60	HCl-99	CLINIC	1	Hepatic	*K. pneumoniae*	CTXM-G1	-	MB
61	HCl-105	CLINIC	Pre-T	Renal	*E. coli*	CTXM-G1	-	NB
62	HCl-106	CLINIC	1	Hepatic	*E. coli*	CTXM-G9	-	NB
63	HCl-109	CLINIC	1	Hepatic	*K. pneumoniae*	CTXM-G1	-	MB
64	HCl-113	CLINIC	1	Renal+pancreatic	*K. pneumoniae*	CTXM-G1	-	MB
65	HCl-114	CLINIC	2	Renal+pancreatic	*K. pneumoniae*	CTXM-G1	-	MB
67	HCl-121	CLINIC	Pre-T	Hepatic	*K. pneumoniae*	CTXM-G1	-	SB
68	HCl-122	CLINIC	4	Renal	*E. coli*	CTXM-G9	-	NB
69	HCl-125	CLINIC	1	Renal	*K. pneumoniae*	CTXM-G1 + OXA-48	-	SB
70	HCl-126	CLINIC	Pre-T	Renal	*E. coli*	CTXM-G1	-	WB
71	HCl-128	CLINIC	5	Renal	*K. pneumoniae*	CTXM-G1	-	MB
72	HCl-129	CLINIC	6	Renal	*K. pneumoniae*	CTXM-G1	-	MB
73	HCl-131	CLINIC	2	Renal	*E. cloacae*	VIM	-	WB
74	HCl-134	CLINIC	2	Hepatic	*K. pneumoniae*	CTXM-G1 + OXA-48	-	SB
75	HCl-135	CLINIC	2	Renal	*K. pneumoniae*	CTXM-G1	-	SB
76	HCl-138	CLINIC	2	Renal	*K. pneumoniae*	CTXM-G1	-	MB
77	HCl-139	CLINIC	2	Renal	*K. pneumoniae*	CTXM-G1	-	SB
78	HCl-140	CLINIC	4	Renal	*E. coli*	CTXM-G1	-	NB
79	HCl-142	CLINIC	Pre-T	Renal	*E. coli*	CTXM-G9	-	NB
80	HCl-147	CLINIC	Pre-T	Renal	*K. pneumoniae*	CTXM-G1	-	NB
81	HCl-148	CLINIC	Pre-T	Renal	*E. coli*	SHV	-	NB
82	HCl-150	CLINIC	4	Renal	*E. coli*	CTXM-G9	-	NB
83	HCl-151	CLINIC	Pre-T	Renal	*K. pneumoniae*	CTXM-G1	-	NB

84	HUC-01	CRUCES	Pre-T	Hepatic	*K. pneumoniae*	SHV	-	MB
85	HUC-08	CRUCES	6	Hepatic	*E. coli*	CTXM-G9	-	NB
86	HUC-10	CRUCES	4	Hepatic	*E. coli*	SHV	-	NB
87	HUC-11	CRUCES	3	Hepatic	*E. coli*	CTXM-G9	-	NB
88	HUC-14	CRUCES	3	Hepatic	*E. coli*	CTXM-G1	-	WB
89	HUC-16	CRUCES	3	Hepatic	*K. pneumoniae*	VIM	-	NB
90	HUC-20	CRUCES	2	Hepatic	*E. coli*	CTXM-G1	-	WB
91	HUC-23	CRUCES	Pre-T	Hepatic	*E. cloacae*	Hiper-AmpC-c	-	NB
93	HUC-25	CRUCES	Pre-T	Hepatic	*E. coli*	CTXM-G9	-	NB
95	HUC-30	CRUCES	3	Hepatic	*K. pneumoniae*	SHV	-	MB
96	HUC-33	CRUCES	3	Renal	*E. coli*	SHV	-	NB
98	HUC-36	CRUCES	3	Hepatic	*E. coli*	CTXM-G9	-	NB
99	HUC-38	CRUCES	Pre-T	Hepatic	*E. coli*	CTXM-G9	-	NB
100	HUC-39	CRUCES	Pre-T	Hepatic	*E. coli*	CTXM-G1	-	NB
101	HUC-40	CRUCES	Pre-T	Hepatic	*E. coli*	CTXM-G9	-	NB
102	HUC-41	CRUCES	Pre-T	Hepatic	*K. pneumoniae*	TEM	-	NB
103	HUC-42	CRUCES	Pre-T	Hepatic	*E. coli*	CTXM-G9	-	NB
104	HUC-43	CRUCES	Pre-T	Hepatic	*E. coli*	CTXM-G9	MRHA	NB
105	HUGM-01	GMARAÑON	Pre-T	Hepatic	*E. coli*	CTXM-G1	MRHA	WB
106	HUGM-04B	GMARAÑON	2	Hepatic	*K. pneumoniae*	CTXM-G1 + OXA-48	-	WB
107	HUGM-05	GMARAÑON	3	Renal	*E. coli*	CTXM-G1	-	MB
108	HUGM-07B	GMARAÑON	3	Renal	*E. coli*	CTXM-G9	-	MB
109	HUGM-08	GMARAÑON	1	Renal	*E. coli*	CTXM-G1	MRHA	WB
110	HUGM-10	GMARAÑON	Pre-T	Renal	*K. pneumoniae*	CTXM-G1 + OXA-48	-	SB
111	HUGM-11	GMARAÑON	Pre-T	Renal	*K. pneumoniae*	CTXM-G1 + OXA-48	-	SB
112	HUGM-15	GMARAÑON	4	Renal	*K. pneumoniae*	OXA-48	-	MB
113	HUGM-21	GMARAÑON	2	Renal	*K. pneumoniae*	OXA-48	-	MB
114	HUGM-22	GMARAÑON	2	Renal	*E. coli*	CTXM-G1 + OXA-48	-	SB
115	HUGM-24	GMARAÑON	Pre-T	Renal	*K. pneumoniae*	CTXM-G1 + OXA-48	-	SB
116	HUGM-29	GMARAÑON	1	Renal	*K. pneumoniae*	CTXM-G1 + OXA-48	-	SB
117	HUGM-38	GMARAÑON	2	Renal	*K. pneumoniae*	CTXM-G1	-	MB
118	HUGM-39	GMARAÑON	1	Renal	*E. coli*	CTXM-G1	MSHA	NB
119	HUGM-40	GMARAÑON	Pre-T	Renal	*K. pneumoniae*	CTXM-G1 + OXA-48	-	MB
120	HUMV-01	HUMV	1	Renal	*E. cloacae*	CTXM-G9	-	NB
121	HUMV-01Hm	HUMV	**Blood**	Renal	*E. cloacae*	CTXM-G9	-	NB
123	HUMV-03	HUMV	1	Renal	*E. coli*	CTXM-G9	-	NB
124	HUMV-07	HUMV	2	Renal	*E. coli*	CTXM-G1	-	NB
125	HUMV-11	HUMV	3	Renal	*E. coli*	TEM	-	NB
126	HUMV-14	HUMV	3	Hepatic	*E. coli*	SHV	-	NB
127	HUMV-15	HUMV	1	Hepatic	*E. coli*	SHV	-	NB

128	HUMV-24	HUMV	4	Hepatic	*E. coli*	CTXM-G1	-	NB
129	HUMV-27	HUMV	3	Renal	*E. coli*	Hiper AmpC-p	-	MB
130	HUMV-26	HUMV	1	Renal	*E. cloacae*	CTXM-G9	-	WB
131	HUMV-32	HUMV	3	Hepatic	*E. cloacae*	Hiper-AmpC-c	-	WB
132	HUMV-33	HUMV	3	Renal	*E. cloacae*	CTXM-G1	-	MB
133	HUMV-34	HUMV	4	Renal	*E. cloacae*	Hiper-AmpC-c	-	NB
134	HUMV-31	HUMV	Pre-T	Hepatic	*E. coli*	CTXM-G9	-	MB
135	HUMV-42	HUMV	3	Renal	*K. pneumoniae*	CTXM-G9	-	MB
136	HUMV-40	HUMV	1	Renal+pancreatic	*E. cloacae*	Hiper-AmpC-c	-	WB
137	HUMV-38	HUMV	Pre-T	Renal	*E. coli*	Hiper AmpC-p	-	MB
138	HUMV-44	HUMV	3	Renal	*K. pneumoniae*	CTXM-G9	-	SB
139	HUMV-49	HUMV	2	Renal	*E. cloacae*	CTXM-G9	-	NB
140	HUMV-50	HUMV	3	Renal	*E. cloacae*	CTXM-G9	-	WB
141	HUMV-59	HUMV	5	Renal	*E. coli*	CTXM-G9	-	WB
142	HUMV-51	HUMV	Pre-T	Hepatic	*E. coli*	CTXM-G9	MRHA	WB
143	HUMV-60	HUMV	3	Hepatic	*E. coli*	SHV	-	NB
145	HUMV-54	HUMV	Pre-T	Renal	*E. coli*	CTXM-G1	MRHA	WB
146	HUMV-66	HUMV	3	Renal	*Enterobacter asburiae*	CTXM-G9	-	WB
148	HUMV-67	HUMV	Pre-T	Renal	*E. cloacae*	Hiper-AmpC-c	-	NB
149	HUMV-68	HUMV	Pre-T	Renal	*E. coli*	CTXM-G1	-	NB
150	HUMV-70	HUMV	2	Renal	*E. coli*	CTXM-G1	-	NB
151	HUMV-72	HUMV	3	Renal	*E. coli*	CTXM-G1	-	NB
154	HUMV-83	HUMV	1	Renal	*E. coli*	SHV	-	NB
155	HUMV-91	HUMV	2	Hepatic	*E. cloacae*	Hiper-AmpC-c	-	WB
156	HUMV-94	HUMV	3	Hepatic	*E. cloacae*	Hiper-AmpC-c	-	NB
158	HUMV-84	HUMV	Pre-T	Hepatic	*K. pneumoniae*	CTXM-G1	-	MB
159	HUMV-90	HUMV	2	Hepatic	*K. pneumoniae*	CTXM-G1	-	SB
160	HUMV-92	HUMV	3	Hepatic	*K. pneumoniae*	CTXM-G1	-	MB
161	HUMV-97	HUMV	4	Hepatic	*K. pneumoniae*	CTXM-G1 + OXA-48	-	MB
163	HUMV-113	HUMV	**Abdominal drainage**	Hepatic	*K. pneumoniae*	CTXM-G1	-	SB
164	HUMV-87	HUMV	1	Renal	*E. coli*	CTXM-G9	-	NB
166	HUMV-111	HUMV	6	Renal	*K. pneumoniae*	CTXM-G1	-	WB
167	HUMV-93	HUMV	2	Renal	*E. aerogenes*	Hiper-AmpC-c	-	NB
168	HUMV-99	HUMV	4	Hepatic	*K. pneumoniae*	CTXM-G1 + OXA-48	-	MB
169	HUMV-103	HUMV	**Bile**	Hepatic	*K. pneumoniae*	CTXM-G1 + OXA-48	-	MB
170	HUMV-86	HUMV	Pre-T	Renal	*E. coli*	SHV	-	NB
171	HUMV-98	HUMV	1	Renal	*K. pneumoniae*	CTXM-G1 + OXA-48	-	NB
172	HUMV-104	HUMV	**Urine**	Renal	*K. pneumoniae*	CTXM-G1 + OXA-48	-	NB
173	HUMV-105	HUMV	**Urine**	Renal	*K. pneumoniae*	CTXM-G1 + OXA-48	-	NB
174	HUMV-117	HUMV	5	Renal	*K. pneumoniae*	CTXM-G1 + OXA-48	-	NB

175	HUMV-121	HUMV	6	Renal	*K. pneumoniae*	CTXM-G1 + OXA-48	-	NB
176	HUMV-101	HUMV	1	Renal	*K. pneumoniae*	CTXM-G1 + OXA-48	-	NB
178	HUMV-115	HUMV	2	Hepatic	*K. pneumoniae*	CTXM-G1	-	MB
179	HUMV-116	HUMV	1	Renal	*E. coli*	CTXM-G9	-	NB
180	HUMV-118	HUMV	3	Hepatic	*K. pneumoniae*	CTXM-G1	-	SB
181	HUMV-119	HUMV	3	Hepatic	*E. coli*	CTXM-G1	MSHA	NB
182	HUMV-122	HUMV	5	Hepatic	*K. pneumoniae*	CTXM-G1	-	SB
183	HUMV-125	HUMV	1	Renal+pancreatic	*E. cloacae*	Hiper-AmpC-c	-	WB
184	HUMV-127	HUMV	6	Renal	*E. coli*	CTXM-G9	-	NB
185	HUMV-128	HUMV	2	Renal	*E. coli*	CTXM-G1	-	NB
186	HUMV-130	HUMV	1	Hepatic	*E. coli*	CTXM-G1	MRHA	NB
187	HUMV-131	HUMV	2	Hepatic	*E. coli*	CTXM-G1	MRHA	NB
188	HUMV-132	HUMV	3	Renal	*E. coli*	CTXM-G1	-	NB
191	HUMV-138	HUMV	1	Hepatic	*K. pneumoniae*	CTXM-G1 + OXA-48	-	NB
192	HUMV-140	HUMV	Pre-T	Renal	*E. coli*	CTXM-G1	-	NB
193	HUMV-143	HUMV	3	Hepatic	*K. pneumoniae*	CTXM-G1 + OXA-48	-	MB
194	HUMV-145	HUMV	4	Hepatic	*E. coli*	CTXM-G1 + OXA-48	-	NB
195	HUMV-146	HUMV	Pre-T	Hepatic	*K. pneumoniae*	CTXM-G1 + OXA-48	-	SB
196	HUMV-148	HUMV	4	Hepatic	*E. coli*	CTXM-G9	-	NB
197	HUMV-152	HUMV	3	Renal	*K. pneumoniae*	CTXM-G1 + OXA-48	-	NB
198	HUMV-155	HUMV	Pre-T	Renal	*E. coli*	CTXM-G1	MRHA	WB
199	HUMV-157	HUMV	**Urine**	Renal	*E. coli*	CTXM-G1	MRHA	WB
200	HUMV-158	HUMV	Pre-T	Hepatic	*E. coli*	CTXM-G1	-	NB
201	HUMV-162	HUMV	2	Hepatic	*K. pneumoniae*	CTXM-G1 + OXA-48	-	SB
203	HUMV-168	HUMV	1	Hepatic	*K. pneumoniae*	CTXM-G1	-	MB
204	HURyC-01	RCAJAL	1	Hepatic	*E. coli*	TEM	MSHA	NB
205	HURyC-04	RCAJAL	1	Hepatic	*K. pneumoniae*	SHV	-	SB
206	HURyC-05	RCAJAL	1	Hepatic	*E. cloacae*	Hiper-AmpC-c	-	WB
208	HURyC-09	RCAJAL	3	Hepatic	*E. coli*	CTXM-G1	-	WB
209	HURyC-08	RCAJAL	2	Hepatic	*K. pneumoniae*	CTXM-G1	-	SB
210	HURyC-12B	RCAJAL	1	Hepatic	*K. pneumoniae*	VIM	-	NB
212	HURyC-15	RCAJAL	1	Hepatic	*E. coli*	CTXM-G8	-	MB
213	HURyC-16	RCAJAL	3	Renal	*E. coli*	CTXM-G1	MSHA	NB
214	HURyC-17	RCAJAL	1	Renal	*E. coli*	CTXM-G1	-	NB
215	HURyC-18	RCAJAL	4	Renal	*K. pneumoniae*	CTXM-G1	-	SB
216	HURyC-21	RCAJAL	1	Hepatic	*K. pneumoniae*	CTXM-G1	-	SB
217	HURyC-23	RCAJAL	5	Hepatic	*E. coli*	CTXM-G1	-	NB

220	HURyC-27	RCAJAL	5	Hepatic	*K. pneumoniae*	CTXM-G1 + OXA-48	-	NB
221	HURyC-28	RCAJAL	3	Renal	*E. coli*	CTXM-G9	MSHA	WB
222	HURyC-29	RCAJAL	1	Renal	*E. coli*	CTXM-G1	-	NB
223	HURyC-32	RCAJAL	5	Renal	*E. coli*	CTXM-G1	-	NB
225	HURyC-34	RCAJAL	1	Renal	*E. coli*	CTXM-G1	-	WB
226	HURyC-35	RCAJAL	1	Hepatic	*E. cloacae*	OXA-48	-	NB
227	HURS-01	RSOFIA	1	Renal	*E. coli*	CTXM-G1	-	NB
228	HURS-04	RSOFIA	**Urine**	Renal	*E. coli*	CTXM-G1	MRHA	WB
229	HURS-05B	RSOFIA	4	Renal+pancreatic	*E. coli*	SHV	MSHA	NB
232	HURS-11	RSOFIA	4	Renal	*E. coli*	SHV	-	WB
233	HURS-15	RSOFIA	4	Renal	*E. aerogenes*	Hiper-AmpC-c	-	WB
234	HURS-17	RSOFIA	4	Renal+pancreatic	*E. coli*	SHV	-	MB
236	HURS-24	RSOFIA	2	Renal	*E. coli*	SHV	MSHA	NB
237	HURS-25	RSOFIA	3	Renal	*E. coli*	CTXM-G1	-	NB
238	HURS-27	RSOFIA	1	Hepatic	*K. pneumoniae*	KPC	-	MB
239	HURS-29	RSOFIA	**Urine**	Hepatic	*K. pneumoniae*	KPC	-	MB
240	HURS-31	RSOFIA	Pre-T	Hepatic	*E. cloacae*	Hiper-AmpC-c	-	NB

[a]Strains from: 12OCTUBRE, Hospital Universitario 12 de Octubre, Madrid; CLINIC, Hospital Clinic, Barcelona; CRUCES, Hospital Universitario Cruces, Baracaldo; GMARAÑON, Hospital Gregorio Marañón, Madrid; RCAJAL, Hospital Universitario Ramón y Cajal, Madrid; RSOFIA, Hospital Universitario Reina Sofía, Córdoba. [b]Date of isolation: Pre-T= pre-transplant sample; 1= 7 days post-transplant; 2= 14 days post-transplant; 3=21 days post-transplant; 4= 30 days post-transplant; 5= 45 days post-transplant; 6= 55 days post-transplant. [c]MSHA, mannose-sensitive hemagglutination; MRHA, mannose-resistant hemagglutination; -, no agglutination of human red blood cells. [d]Biofilm formation: NB, non-biofilm; WB, weak; MB, moderate; SB, strong.

Supplementary Table S2

Primers used in the detection of antimicrobial resistance genes and expected amplicon sizes

**Resistance**	**Gene**	**DNA sequence (5´–3´)**	**Amplicon size (bp)**	**Annealing Temperature (ºC)**	**Reference**
β-lactams	*bla*$_{TEM}$	ATGAGTATTCAACATTTCCG	867	55	[1]
		CTGACAGTTACCAATGCTTA			
	bla$_{SHV}$	GGGTTATTCTTATTTGTCGC	930		[1]
		TTAGCGTTGCCAGTGCTC			
Cephalosporins	*bla*$_{CTXM}$	ATGTGCAGYACCAGTAARGT	593	55	[2]
		TGGGTRAARTARGTSACCAGA			
	bla$_{CTXM\text{-}group1}$	ATGGTTAAAAAATCACTGCG	912	55	[3]
		TTACAAACCGTCGGTGAC			
	bla$_{CTXM\text{-}group9}$	ATGGTGACAAAGAGAGTGCAAC	876	55	[3]
		TTACAGCCCTTCGGCGATG			
	bla$_{CTXM\text{-}group8}$	TCGCGTTAAGCGGATGATGC	666	55	[4]
		AACCCACGATGTGGGTAGC			
Cephamycinases	*bla*$_{ACC}$	AACAGCCTCAGCAGCCGGTTA	346	64	[5]
		TTCGCCGCAATCATCCCTAGC			
	bla$_{CIT}$	TGGCCAGAACTGACAGGCAAA	462		[5]
		TTTCTCCTGAACGTGGCTGGC			
	bla$_{DHA}$	AACTTTCACAGGTGTGCTGGGT	405		[5]
		CCGTACGCATACTGGCTTTGC			
	bla$_{MOX}$	GCTGCTCAAGGAGCACAGGAT	520		[5]
		CACATTGACATAGGTGTGGTGC			
	bla$_{EBC}$	TCGGTAAAGCCGATGTTGCGG	302		[5]
		CTTCCACTGCGGCTGCCAGTT			
	bla$_{FOX}$	AACATGGGGTATCAGGGAGATG	190		[5]
		CAAAGCGCGTAACCGGATTGG			
Carbapenems	*bla*$_{OXA\text{-}48}$	TGCGTGTATTAGCCTTATCG	784	60	[6]
		TTTTTCCTGTTTGAGCACTTC			
	bla$_{IMP}$	GAAGGCGTTTATGTTCATAC	586	55	[7]
		GTAAGTTTCAAGAGTGATGC			
	bla$_{KPC}$	CATTCAAGGGCTTTCTTGCTGC	538	60	[8]
		ACGACGGCATAGTCATTTGC			
	bla$_{NDM}$	CCATGCGGGCCGTATGAGTGATTG	700	60	[9]
		TCGCGAAGCTGAGCACCGCATTAG			
	bla$_{VIM}$	ATGGTGTTTGGTCGCATATC	510	60	[10]
		TGGGCCATTCAGCCAGATC			

References

[1] Rasheed JK, Jay C, Metchock B, Berkowitz F, Weigel L, Crellin J, Steward C, Hill B, Medeiros AA, Tenover FC. Evolution of extended-spectrum beta-lactam resistance (SHV-8) in a strain of *Escherichia coli* during multiple episodes of bacteremia. Antimicrob Agents Chemother. 1997 Mar;41(3):647-53.

[2] Pagani L, Dell'Amico E, Migliavacca R, D'Andrea MM, Giacobone E, Amicosante G, Romero E, Rossolini GM. Multiple CTX-M-type extended-spectrum beta-lactamases in nosocomial isolates of *Enterobacteriaceae* from a hospital in northern Italy. J Clin Microbiol. 2003 Sep;41(9):4264-9.

[3] Ruiz del Castillo B, Vinué L, Román EJ, Guerra B, Carattoli A, Torres C, Martínez-Martínez L. Molecular characterization of multiresistant *Escherichia coli* producing or not extended-spectrum β-lactamases. BMC Microbiol. 2013 Apr 16;13:84

[4] Woodford N, Fagan EJ, Ellington MJ. Multiplex PCR for rapid detection of genes encoding CTX-M extended-spectrum (beta)-lactamases. J Antimicrob Chemother. 2006 Jan;57(1):154-5.

[5] Perez-Perez, F.J. Hanson, N.D. Detection of plasmid-mediated AmpC beta-lactamase genes in clinical isolates by using multiplex PCR. Journal of clinical microbiology (2002) 40, 2153-2162.

[6] Oteo J, Saez D, Bautista V, Fernández-Romero S, Hernández-Molina JM, Pérez-Vázquez M, Aracil B, Campos J; Spanish Collaborating Group for the Antibiotic Resistance Surveillance Program. Carbapenemase-producing *Enterobacteriaceae* in Spain in 2012. Antimicrob Agents Chemother. 2013 Dec;57(12):6344-7.

[7] Miró E, Agüero J, Larrosa MN, Fernández A, Conejo MC, Bou G, González-López JJ, Lara N, Martínez-Martínez L, Oliver A, Aracil B, Oteo J, Pascual A, Rodríguez-Baño J, Zamorano L, Navarro F. Prevalence and molecular epidemiology of acquired AmpC β-lactamases and carbapenemases in *Enterobacteriaceae* isolates from 35 hospitals in Spain. Eur J Clin Microbiol Infect Dis. 2013 Feb;32(2):253-9.

[8] Dallenne C, Da Costa A, Decré D, Favier C, Arlet G. Development of a set of multiplex PCR assays for the detection of genes encoding important beta-lactamases in Enterobacteriaceae. J Antimicrob Chemother. 2010 Mar;65(3):490-5.

[9] Oteo J, Domingo-García D, Fernández-Romero S, Saez D, Guiu A, Cuevas O, Lopez-Brea M, Campos J. Abdominal abscess due to NDM-1-producing *Klebsiella pneumoniae* in Spain. J Med Microbiol. 2012 Jun;61(Pt 6):864-7.

[10] Poirel L, Naas T, Nicolas D, Collet L, Bellais S, Cavallo JD, Nordmann P. Characterization of VIM-2, a carbapenem-hydrolyzing metallo-b-lactamase and its plasmid- and integron-borne gene from *Pseudomonas aeruginosa* clinical isolate in France. Antimicrob Agents Chemoter. 2000 Apr;44(4): 891-7
